# Current Clinical Treatments and Challenges for Cardiovascular Diseases: From Therapeutic Expansion to Individualized Care

**DOI:** 10.3390/jcm15176607

**Published:** 2026-08-27

**Authors:** Christos Kourek, Georgios Bouras

**Affiliations:** 1 Medical School of Athens, National and Kapodistrian University of Athens, 15772 Athens, Greece; chris.kourek.92@gmail.com; 2 Yale School of Medicine, Yale University, New Heaven, CT 06510, USA

This Editorial closes the Special Issue “Current Clinical Treatments and Challenges for Cardiovascular Diseases”, which was conceived to examine contemporary cardiovascular care at the point where therapeutic progress meets persistent clinical uncertainty [1,2,3,4,5,6]. Cardiovascular medicine has entered an era of rapidly expanding procedural, pharmacological, imaging, and biomarker-based options, yet cardiovascular disease remains the leading contributor to global morbidity and mortality [7,8]. The central problem is therefore no longer simply whether an effective treatment exists. Increasingly, it is how to select the right treatment for the right patient, at the right time, while accounting for biological heterogeneity, competing risks, procedural complexity, comorbid disease, and the gap between trial efficacy and everyday effectiveness. The six contributions to this Special Issue approach that problem from different directions, infectious myocarditis, transcatheter and surgical valve intervention, postoperative atrial fibrillation (POAF), bleeding prevention during antithrombotic therapy, and cardiovascular–kidney–metabolic syndrome (CKMS), but together they support a common message: better cardiovascular outcomes depend as much on individualized clinical interpretation as on therapeutic innovation itself [1,2,3,4,5,6].

The contribution by Zouganeli et al. illustrates this principle in a setting in which diagnostic recognition precedes treatment [1]. *Campylobacter jejuni* myocarditis is uncommon, but its rarity is precisely what makes it clinically instructive. The reported patient was a young man with febrile diarrhea and acute chest pain, elevated cardiac troponin, and myocardial inflammation documented by cardiac magnetic resonance (CMR), despite limited findings on conventional testing. The accompanying literature synthesis identified a striking predominance in young men and emphasized the recurrent temporal association between gastrointestinal infection and myocardial injury [1]. This report is clinically relevant beyond *Campylobacter* infection itself. Current myocarditis practice increasingly relies on a risk-based diagnostic pathway in which CMR plays a central role, while endomyocardial biopsy is reserved for selected scenarios in which histological or etiological clarification is expected to alter management [9,10,11,12,13]. The important lesson is not to over-attribute causality to a positive extracardiac microbiological result—the absence of myocardial pathogen demonstration remains a limitation—but to maintain etiological breadth when a young patient presents with chest pain, troponin elevation, and a recent infectious syndrome. Early recognition, appropriate imaging, targeted microbiological investigation, and structured follow-up can prevent a rare diagnosis from becoming a missed diagnosis.

Aortic stenosis represents a different challenge: not therapeutic scarcity, but therapeutic abundance. Transcatheter aortic valve implantation (TAVI) has transformed the treatment of severe aortic stenosis and has progressively moved into lower-risk and younger populations [14,15,16,17,18,19,20,21]. As indications expand, however, the relevant question becomes more demanding than “TAVI or surgery?”. Contemporary decision-making must integrate age, life expectancy, anatomy, vascular access, coronary disease, concomitant valve or aortic pathology, prosthesis durability, future coronary access, feasibility of reintervention, patient preference, and institutional expertise. The 2025 ESC/EACTS guidelines reinforce this lifetime-management approach and the central role of the Heart Team [14]. Two contributions to this Special Issue are particularly valuable because they interrogate this decision from complementary real-world perspectives [2,3].

Sticchi et al. evaluated sex-specific outcomes in 3353 patients treated with contemporary self-expandable transcatheter valves [2]. Women comprised 64.5% of the cohort and, after propensity matching, experienced a higher one-year composite rate of death or stroke than men (9.4% vs. 6.0%), driven mainly by stroke (2.8% vs. 1.2%), while overall mortality was similar. Major vascular complications, bleeding, and acute kidney injury were also more frequent among women [2]. These data add to a literature in which sex-related TAVI outcomes have not been uniform [22,23]. Their practical implication is not that women derive less value from TAVI, but that sex should not be treated as a passive demographic covariate. Smaller vascular dimensions, bleeding susceptibility, renal vulnerability, and potentially different embolic risk profiles should inform procedural planning. The resulting strategy is concrete: meticulous vascular assessment, appropriately sized access systems, attention to contrast burden and hemodynamics, and individualized consideration of stroke-prevention measures [2,24]. Comparable survival despite greater procedural vulnerability also argues against therapeutic exclusion and instead for procedural adaptation.

Ranucci et al. address the equally important tension between randomized evidence and real-world effectiveness [3]. In the OUTSTANDING ITALY study, patients aged 65–80 years undergoing TAVI or surgical aortic valve replacement (SAVR) were identified from administrative databases in Lombardy and Puglia and compared after propensity matching. At the end of follow-up, mortality was substantially lower after SAVR in both regions; 24.6% versus 47.2% in Lombardy and 18.1% versus 44.1% in Puglia [3]. These observations contrast with randomized trials in selected low-risk populations, in which transcatheter and surgical strategies have shown broadly comparable intermediate- and longer-term clinical outcomes [16,17,18,19,20,21]. The discrepancy should not be resolved by choosing one evidence source and dismissing the other. Randomized trials maximize internal validity but enroll selected patients and are often performed in highly experienced centers; observational analyses capture routine practice but remain susceptible to residual confounding, referral patterns, coding limitations, and unmeasured differences in frailty, ventricular function, surgical risk, or anatomy. The value of the OUTSTANDING ITALY study lies precisely in making this gap visible [3]. For patients in the intermediate-age range, particularly those with long life expectancy, treatment should remain a lifetime strategy rather than an isolated procedural choice. Real-world registries, contemporary randomized follow-up, and studies of durability and reintervention should therefore be interpreted together rather than competitively [19,20,21,25,26].

The contribution by Fudulu et al. shifts the focus from selecting an intervention to understanding what happens around it [4]. POAF remains one of the most frequent complications after cardiac surgery and is associated with greater resource use and adverse downstream outcomes [27,28,29,30]. In a post hoc analysis of a randomized-trial database, the investigators examined ventricular adenine nucleotide metabolism in 88 patients undergoing coronary artery bypass grafting (CABG) or aortic valve replacement. POAF occurred in 27 patients (31%), and age and aortic cross-clamp time emerged as significant clinical predictors. Among CABG patients who developed POAF, ATP/ADP and ATP/AMP ratios fell after reperfusion, supporting a possible link between intraoperative ischemia, energetic disturbance, and postoperative arrhythmogenesis [4]. The analysis is appropriately hypothesis-generating, particularly because the metabolic signal was not reproduced in the valve-surgery subgroup and atrial tissue was unavailable. Nevertheless, it provides an important translational direction. Age is not modifiable; cross-clamp duration, myocardial protection, and ischemia–reperfusion exposure are. Mechanistic studies are most useful when they identify variables that can eventually be incorporated into prevention. In this context, the study connects cellular energetics with an everyday surgical complication and reinforces continued efforts to optimize cardioprotection and perioperative AF prevention [27,31,32].

Modern cardiovascular therapy also creates treatment-related risk. Antiplatelet and anticoagulant strategies prevent ischemic and thromboembolic events, but bleeding—particularly gastrointestinal bleeding—may offset part of that benefit in vulnerable patients [33,34,35]. The LORA-HBR study by Kang et al. addresses this competing-risk problem [5]. In a prospective, multicenter, single-arm study of 909 South Korean patients with high-bleeding-risk features receiving chronic antithrombotic therapy, low-dose rabeprazole (5 mg daily) was associated with no significant upper gastrointestinal bleeding or symptomatic peptic ulcer disease during follow-up, high adherence, and low rates of cardiovascular events and treatment discontinuation [5]. The findings are consistent with the established gastroprotective effect of proton pump inhibitor co-therapy in patients receiving antithrombotic treatment [36,37,38], while exploring whether protection can be achieved with a lower maintenance dose in a population in whom bleeding risk is clinically important. At the same time, the interpretation should remain proportional to the design: the absence of a control group, the lower-than-expected event rate, the stable chronic-phase profile of many participants, and the absence of primary bleeding events limit causal inference [5]. Rather than closing the question, the study defines the next one more precisely: which patients need gastroprotection, at what dose, and for how long, to preserve ischemic benefit while minimizing gastrointestinal and long-term treatment-related risk?

The narrative review by Sławiński et al. broadens the Special Issue from treatment of established cardiovascular disease to earlier identification of systemic risk [6]. The CKMS framework recognizes that cardiovascular disease, chronic kidney disease, obesity, diabetes, and related metabolic abnormalities are not parallel comorbidities but interacting components of a shared pathophysiological continuum [39,40]. This shift has practical consequences. Laboratory assessment is no longer limited to confirming an isolated diagnosis; it can be used to stage risk and identify subclinical organ involvement before overt cardiovascular events occur. The authors emphasize the complementary roles of estimated glomerular filtration rate, cystatin C, urinary albumin-to-creatinine ratio, natriuretic peptides, high-sensitivity troponin, glycemic indices, and lipid parameters, and propose the integration of renal function, albuminuria, and cardiac stress into longitudinal CKMS assessment [6]. This approach is consistent with contemporary CKD and diabetes guidance, in which albuminuria, renal function, cardiovascular status, and metabolic phenotype increasingly determine the intensity and choice of organ-protective therapy [41,42]. Importantly, biomarkers are only useful when they change management. Future CKMS research should therefore move beyond associations toward prospective testing of whether biomarker-guided staging improves treatment implementation, clinical outcomes, quality of life, and cost-effectiveness.

Taken together, the contributions to this Special Issue reveal several recurring challenges in contemporary cardiovascular medicine. First, external validity matters. The expansion of TAVI demonstrates that results from randomized trials and routine practice must be continuously reconciled rather than assumed to be interchangeable [2,3]. Second, heterogeneity matters. Sex-specific procedural risk, age, renal vulnerability, bleeding propensity, metabolic phenotype, and infectious context can materially alter the interpretation of an otherwise standardized treatment pathway [1,2,5,6]. Third, mechanisms matter when they identify actionable targets. The ventricular energetic changes associated with POAF are valuable because they generate testable hypotheses concerning myocardial protection and ischemic exposure [4]. Fourth, competing risk matters. A treatment that reduces one cardiovascular event may create another clinically relevant hazard, making prevention of bleeding and other adverse effects part of cardiovascular efficacy rather than an ancillary consideration [5]. Finally, multidisciplinary care is no longer optional. Heart Team assessment in valvular disease and integrated cardiovascular–renal–metabolic care are manifestations of the same principle: complex patients cannot be optimally managed within isolated organ or procedural silos [6,14,39,40,41,42].

The next phase of cardiovascular progress will therefore depend less on adding therapies indiscriminately and more on improving selection, sequencing, safety, and implementation. Future studies should deliberately include populations that are under-represented in pivotal trials; report sex-specific and phenotype-specific outcomes; link mechanistic biomarkers to clinically actionable interventions; prioritize long-term, patient-centered outcomes; and use pragmatic designs to test whether evidence remains effective when transferred into routine care. Registries should complement randomized trials, not compete with them, while diagnostic advances should be judged by whether they improve decisions rather than by discrimination alone.

In closing this Special Issue, the six published contributions collectively demonstrate the breadth of current cardiovascular care and, more importantly, the nature of its remaining challenges [1,2,3,4,5,6]. From recognizing an uncommon infectious myocarditis to choosing between transcatheter and surgical valve replacement, reducing postoperative arrhythmia, protecting patients from antithrombotic-related gastrointestinal harm, and identifying cardiovascular–renal–metabolic risk before overt organ failure, the common objective is the same: to translate expanding evidence into safer and more individualized clinical care. We thank all authors and reviewers whose work shaped this collection and hope that these articles will stimulate further studies directed not only at what can be treated, but at how cardiovascular treatment can be better matched to the patient in front of us.
